# Paraprobiotic derived from *Bacillus velezensis* GV1 improves immune response and gut microbiota composition in cyclophosphamide-treated immunosuppressed mice

**DOI:** 10.3389/fimmu.2024.1285063

**Published:** 2024-02-22

**Authors:** Hyo-Jun Lee, My Thi Hoa Tran, Minh Ha Le, Elsa Easter Justine, Yeon-Ju Kim

**Affiliations:** Graduate School of Biotechnology, and College of Life Science, Kyung Hee University, Yongin-si, Gyeonggi-do, Republic of Korea

**Keywords:** paraprobiotics, Bacillus velezensis GV1, immunoregulation, gut mirobiota, cyclophosphamide

## Abstract

Paraprobiotics that benefit human health have the capacity to modulate innate and adaptive immune systems. In this study, we prepared the paraprobiotic from *Bacillus velezensis* GV1 using the heat-killing method and investigated its effects on immunity and gut microbiota *in vitro* and *in vivo*. The morphology of inactivated strain GV1 was observed using scanning electron microscopy. Treatment with GV1 promoted nitric oxide production and augmented cytokine (IL-6, IL-1β, and TNF-α) expression and secretion in RAW 264.7 macrophages. Moreover, the strain GV1 could alleviate cyclophosphamide monohydrate (CTX)-induced immunosuppression by reversing spleen damage and restoring the immune organ index, as well as by increasing the expression of immune-related cytokines (TNF-α, IL-1β, IFN-γ, and IL-2) in the spleen and thymus, respectively. Furthermore, GV1 treatment dramatically healed the CTX-damaged colon and regulated gut microbiota by increasing the relative abundance of beneficial bacterial families (*Lactobacillaceae*, *Akkermansiaceae*, and *Coriobacteriaceae*) and decreasing that of harmful bacterial families (*Desulfovibrionaceae*, *Erysipelotrichaceae*, and *Staphylococcaceae*). Thus, the heat-killed GV1 can be considered a potential immunoregulatory agent for use as a functional food or immune-enhancing medicine.

## 1 Introduction

The immune system of an organism is responsible for protecting against pathogens and maintaining homeostasis for survival (1). Dysfunctional immune responses in the human body due to factors such as the environment, genetics, age, nutrition, and stress can result in immunodeficiency disorders (2). Recently, many immunopotentiation agents have been used to improve immune responses and enhance disease resistance. However, they frequently cause a variety of side effects, including gastrorrhagia, severe neurological lesions, anemia, and colic (3). Research has shown that the use of natural products from fungi, microorganisms, plants, and animals to regulate the immune system is safe and does not cause side effects (4). Therefore, natural products are potential sources of compounds that can improve the immune system without triggering unwanted responses.

Probiotics are known to provide numerous benefits to human health as living microorganisms. Particularly, they are recognized for their crucial features such as immune system enhancement, prevention of gastrointestinal infections, and protection against oxidative stress (5). However, along with these benefits, probiotics may induce side effects in specific population groups, and issues related to their viability, stability, and sensitivity to storage conditions are also associated (6, 7). Alternatives such as prebiotics, paraprobiotics, and postbiotics have been proposed to address these shortcomings of probiotics.

Paraprobiotics, also known as non-viable microbial cells or inactivated probiotics, have garnered recognition for their ability to confer health benefits when administered in suitable quantities. They offer safety advantages by addressing concerns related to viability, survival challenges, and safety considerations regarding microbial movement and infection (8). Recent research endeavors have been initiated to address the limitations associated with probiotics across various domains, including the food industry and therapeutic applications, through the application of paraprobiotics (9, 10). Studies have illustrated that paraprobiotics manifest anti-inflammatory effects, mitigating conditions like colitis, and contribute to enhanced skin moisturization, thereby preventing wrinkle formation (11–15). Numerous reports indicate that probiotics subjected to heat-killing, a potential method for creating paraprobiotics, have a significant impact on immunomodulation (12, 13). Furthermore, recent studies indicate that paraprobiotics manufactured based on this premise exert an influence on immune responses in macrophages and splenocytes (16, 17). Additionally, these investigations propose that peptidoglycan, lipoteichoic acid, and wall teichoic acid obtained from gram-positive microorganisms play a role in immune regulation (18–20). Consequently, these findings underscore the immunomodulatory efficacy of compositions derived from gram-positive paraprobiotics.

*Bacillus velezensis* is a gram-positive, spore-forming bacterium commonly found in soil, plant roots, and fermented foods (21). Spore-forming bacteria, widely employed in medical, veterinary, and more recently in the food industry, exhibit significant potential due to their high resistance and exceptional stability under processing conditions (22). Particularly within the realms of food and fermentation industries, the strain *B. velezensis* has gained notable recognition for its crucial role in ensuring safety and outcompeting rival microorganisms (23, 24). Although bacterial species producing heat-resistant spores are fortunately non-pathogenic, they can lead to food product spoilage (25). Hence, this study explores the immune-enhancing effects of paraprobiotics *Bacillus velezensis* GV1, with an emphasis on the safety of heat-treated strains, for potential applications in health functional foods and food industry, focusing on immune regulation and gut microbiota modulation.

## 2 Materials and methods

### 2.1 Materials

De Man, Rogosa and Sharpe (MRS) broth was obtained from Becton Dickinson & Company (B.D., New Jersey, USA). DMEM medium, penicillin-streptomycin (PS), and fetal bovine serum (FBS) were purchased from GenDEPOT (Katy, TX, USA). Macrogen (Seoul, Republic of Korea) designed all the primers. Live/Dead cell viability assay kits were provided by Thermo Fisher Scientific, USA. Eosin Y Alcoholic was obtained from BBC Biochemical (Mount Vernon, WA, USA). Cyclophosphamide monohydrate (CTX), 3-(4, 5-dimethylthiazol-2-yl)-2, 5-diphenyltetrazolium bromide (MTT), and levamisole hydrochloride (LMS) were purchased from Sigma-Aldrich (St. Louis, MO, USA).

### 2.2 Bacterial strain and heat treatment

The strain GV1 was isolated from ginseng vinegar and identified by 16S rRNA sequencing using four primers (27F, 1492R, 518F, and 800R). The NCBI accession number (16S rRNA gene sequence) of GV1 was OP658964. Additionally, GV1 was deposited in the Korean Collection for Type Cultures (KCTC) under the accession number KCTC 15222BP. The strain was cultured in 1 L of MRS broth at 37°C for 24 h. After this incubation process, the bacterial cells were collected by centrifugation at 4000 rpm for 10 min. The bacterial cell pellet was washed thrice with phosphate-buffered saline (pH 7.4) and re-suspended such that the optical density of the suspension was OD_600_ 1.0. Heat treatment was conducted at 121°C for 15 min. Then, inactivated bacterial cells were freeze-dried for further experiments.

### 2.3 Scanning electron microscope

The strain GV1 was inoculated into MRS broth and cultured for 24 h at 37°C. Heat-killed GV1 was prepared as described above. Briefly, live and heat-killed bacterial cells were collected by centrifugation and pre-fixed with glutaraldehyde (2.5% *v*/*v*) for 2 h. Then, the samples were washed with 0.05 M sodium cacodylate buffer and treated with 1% osmium tetroxide for 1 h. After dehydration using ethanol (in a stepwise elevation from 30% to 50%, 70%, 80%, 90%, and 100%), the samples were treated with hexamethyldisilazane and metallized using platinum. Observations were performed under a SIGMA Field-Emission Scanning Electron Microscope.

### 2.4 Cell culture and viability assay

RAW 264.7 cells were purchased from the Korean Cell Line Bank (KCLB, Korea). The macrophages were maintained in DMEM with 10% FBS and 1% PS inside a 5% CO_2_ incubator at 37°C. After 90% confluency, the cells were seeded in 96-well plates (2 × 10^5^ cells/mL). Then, the cells were treated with different concentrations of GV1 (0.5, 1, and 2 μg/mL) and a positive control, LPS (1 μg/mL), for 24 h. After removing the supernatant, MTT solution (100 μL at 0.5 mg/mL) was added and the plates were incubated for 3 h; then, DMSO was used to dissolve the formazan crystals. The absorbance at 560 nm was measured using a microplate reader (FilterMax F5, Molecular Devices, San Francisco, CA, USA).

### 2.5 Live/dead fluorescence assay

The cytotoxicity of GV1 toward RAW 264.7 cells was assessed using a Live/Dead staining kit (L-3224, Invitrogen, Carlsbad, CA, USA). The macrophages were sub-cultured in small cell culture dishes overnight and then incubated with GV1 (0.5, 1, and 2 μg/mL) and LPS (1 μg/mL). Following 24 h of treatment, calcein AM and ethidium homodimer-1 dyes were mixed with fresh media and added to the cells, which were thereafter incubated for 30 min in the dark. Then, the cells were observed using a Leica DMLS Clinical Microscope (Leica, Wetzlar, Germany).

### 2.6 Measurement of NO

After overnight incubation in 96-well plates (2 × 10^5^ cells/mL), macrophages were treated with GV1 (0.5, 1, and 2 μg/mL) and LPS (1 μg/mL) for 24 h. The culture supernatants (100 μL) were transferred to new plates and 100 μL of Griess reagent was added for NO detection. The absorbance at 570 nm was measured using a microplate reader (FilterMax F5).

### 2.7 Quantitative real-time polymerase chain reaction

Total mRNA was extracted from RAW 264.7 cells and mouse organs in accordance with the TRIzol reagent kit instructions (Invitrogen). The amfiRivert cDNA Synthesis Platinum Enzyme Mix (GenDEPOT) was then used to reverse-transcribe total RNA. AmfiSure qGreen Q-PCR Master Mix (GenDEPOT) was used to perform qRT-PCR using 50 ng of cDNA in a 20 μL reaction volume. The sequences of primers are shown in **Supplementary Table S1**.

### 2.8 Enzyme-linked immunosorbent assay

For *in vitro* experiments, RAW 264.7 cells were seeded in 96-well plates and incubated with GV1 and LPS. The culture supernatants were collected to investigate the production of TNF-α, IL-1β, and IL-6 using an ELISA kit (R&D Systems, Minneapolis, MN, USA), based on the manufacturer’s instructions.

For *in vivo* experiments, spleens of ICR mice in each treatment group were harvested and washed with PBS. Spleen tissues (100 mg) were homogenized and transferred to saline tubes. The tubes were centrifuged at 10000 rpm for 5 min and the supernatants were collected. The contents of TNF-α, IL-1β, IL-6 in the samples were determined using ELISA kits (R&D Systems).

### 2.9 Animal experiments

Male ICR mice (six weeks old) weighing 25 ± 1 g were obtained from OrientBio (Seongnam, Republic of Korea) and housed under stable condition (temperature: 23 ± 2°C, humidity: 50% ± 10%, light/dark cycle: 12 h). This study was approved by the Animal Care and Use Guidelines of Kyung Hee University (KHGASP-23-046). The mice were separated into 6 groups (8 mice per group): Control (CON) group (normal saline), only CTX-treated group, CTX + GV1 (5 mg/kg) group, CTX + GV1 (10 mg/kg) group, CTX + GV1 (20 mg/kg) group, and CTX + LMS (40 mg/kg) group (positive control). For immunosuppression, all groups of animals were injected with CTX (80 mg/kg) for 3 days before GV1 and LMS treatment, except the CON group, which received saline only. GV1 or levamisole hydrochloride (LMS) was orally administered to the mice once daily by gavage for a period of 20 days. Thereafter, the mice were sacrificed to obtain their spleens, guts, and thymus glands for further experiments. The spleens and thymus glands were weighed to calculate the organ index as follows:


$$Index=\frac{Weight\;of\;spleen\;or\;thymus\;\left(g\right)}{Body\;weght\;\left(g\right)}$$

### 2.10 Histopathological analysis of spleen and colon

Immediately after sampling, spleen and colon tissues were fixed with 10% formalin buffer solution and embedded in paraffin for hematoxylin and eosin (HE) staining. Then, the tissue sections were observed under a microscope.

### 2.11 High-throughput sequencing

The genomic DNA of mice feces in the CON, CTX, and GV1 (20 mg/kg) groups was extracted using an E.Z.N.A.^®^ Soil DNA Kit (Omega Biotek, Norcross, GA, USA) following the manufacturer’s protocol. The sequencing procedure was identical to that followed in our previous study (5).

### 2.12 Statistical analysis

Data were expressed as means ± standard deviations or standard errors for *in vitro* or *in vivo* experiments, respectively. All experiments were carried out in triplicate. Statistical comparisons between groups were conducted using Student’s *t*-test and statistical significance was set at different levels (*p*< 0.05, *p*< 0.01, and *p*< 0.001). An analysis of variance (ANOVA) followed by Duncan’s test was employed to evaluate the statistical significance between groups (SPSS 29.0). Different letters presented in tables and figures (a, b, c, d, e) were regarded as statistical significance (*p* < 0.05).

## 3 Results

### 3.1 Field-emission scanning electron microscope

**Figure 1:**
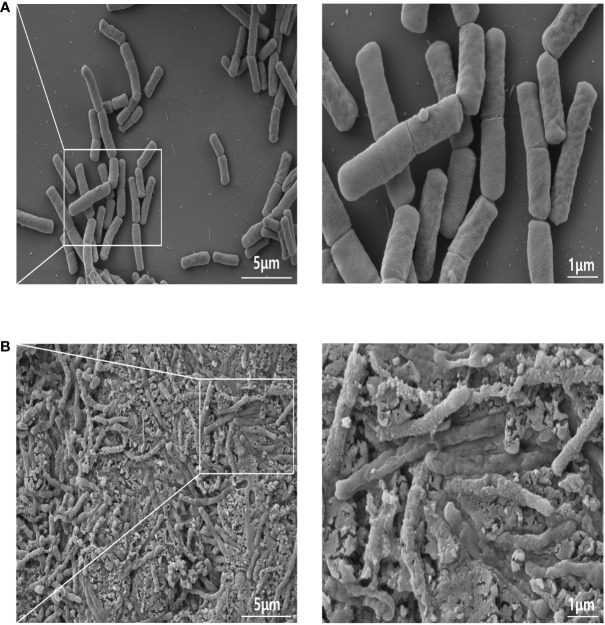
FE-SEM images of GV1: A comparative analysis of live and heat-killed morphology. **(A)** Live GV1; **(B)** Heat-Killed GV1. (10K X, Scale bar = 5μm; 30K X, Scale bar = 1μm).

Generally, the surface of heat-killed bacterial cells was rougher and uneven compared with that of viable cells. **Figures 1A**, **B** show representative FE-SEM images of both live and heat-treated GV1 (at 121°C for 15 min and, consequently, freeze-dried), respectively. There were obvious signs of damage on the surfaces of heat-treated bacterial cells, unlike the untreated cells, indicating that heat treatment inactivated GV1.

### 3.2 Cytotoxicity of GV1 toward RAW 264.7 cells

**Figure 2:**
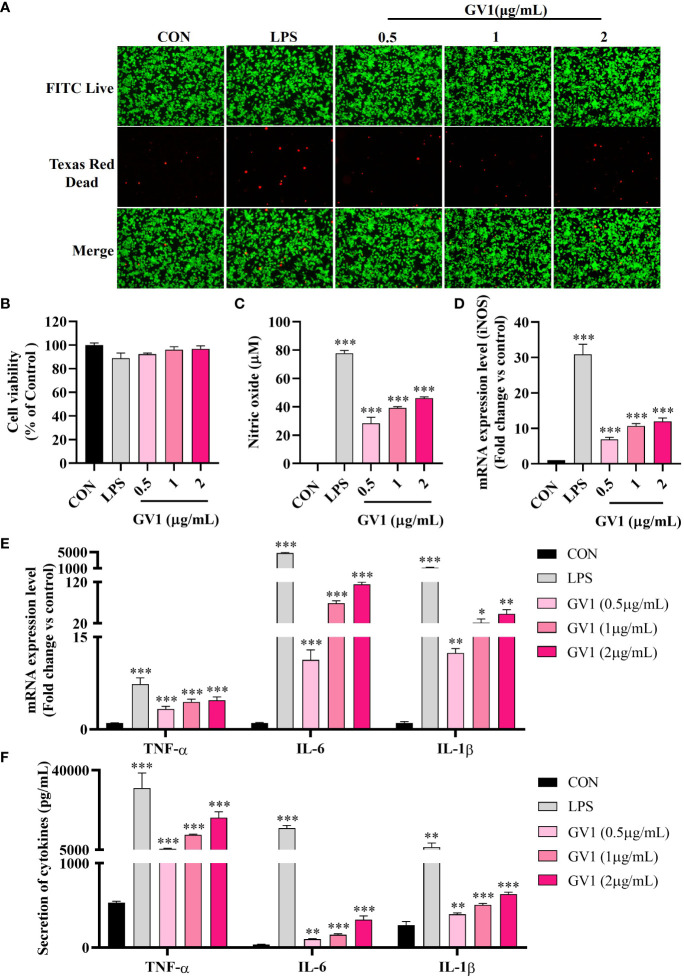
Macrophage responses to GV1: Cytotoxicity and immunity enhancement. **(A)** Live/Dead staining enhancement; **(B)** Cell viability; **(C)** NO production; Expression of mRNA **(D)** *iNOS*, **(E)** *TNF-α*, *IL-6*, and *IL-1β*; **(F)** Secretion of cytokines TNF-α, IL-6, and IL-1β. All data are presented as means ± S.D. *p< 0.05, **p< 0.01, ***p< 0.001 *vs*. CON group.

GV1 cytotoxicity toward RAW 264.7 cells was examined using an MTT assay and Live/Dead staining. The results (Live/Dead staining: **Figure 2A**; MTT assay: **Figure 2B**) revealed that treatment with GV1 (0.5, 1, and 2 μg/mL) had no impact on the viability of macrophages. However, LPS (1 μg/mL) exhibited a slight cytotoxic effect on RAW 264.7 cells.

### 3.3 Effect of GV1 on NO production and inducible *nitric oxide synthase* Expression in RAW 264.7 cells

NO exerts significant antimicrobial, anticancer, and immunomodulation effects but can also damage tissues (26). Hence, we examined the effect of GV1 on NO production in murine macrophages. As shown in **Figure 2C**, GV1 dose-dependently enhanced NO production at 28.3 ± 4.2, 39.2 ± 0.9, and 46.1 ± 0.8 µM at concentration 0.5, 1, and 2 μg/mL, respectively. NO production was mediated by *iNOS*, which is also associated with immune responses (27). Cell stimulation by several agents such as LPS induces *iNOS* expression, thus increasing NO production (28). *iNOS* levels were determined using qRT-PCR (**Figure 2D**); GV1 significantly induced *iNOS* mRNA expression. These findings suggest that GV1 can stimulate RAW 264.7 cells by elevating the expression of *iNOS* and enhancing NO production.

### 3.4 Effect of GV1 on immune-related cytokines in RAW 264.7 cells

It is well known that macrophages modulate adaptive and innate immune systems by releasing immune-related cytokines such as TNF-α, IL-6, and IL-1β (29). Several studies have shown that LPS highly stimulates the expression of *TNF-α*, *IL-6*, and *IL-1β* mRNA (**Figure 2E**) (30, 31). Treatment with GV1 enhanced the expression levels of such cytokine mRNA in a dose-dependent manner (**Figure 2E**). ELISA analysis confirmed that GV1 markedly promoted TNF-α, IL-6 and IL-1β secretion (**Figure 2F**). These findings suggest that GV1 can increase the expression and secretion of immune-related cytokines in murine macrophages.

### 3.5 Effect of GV1 on body weight and immune-related organs in CTX-treated mice

**Figure 3:**
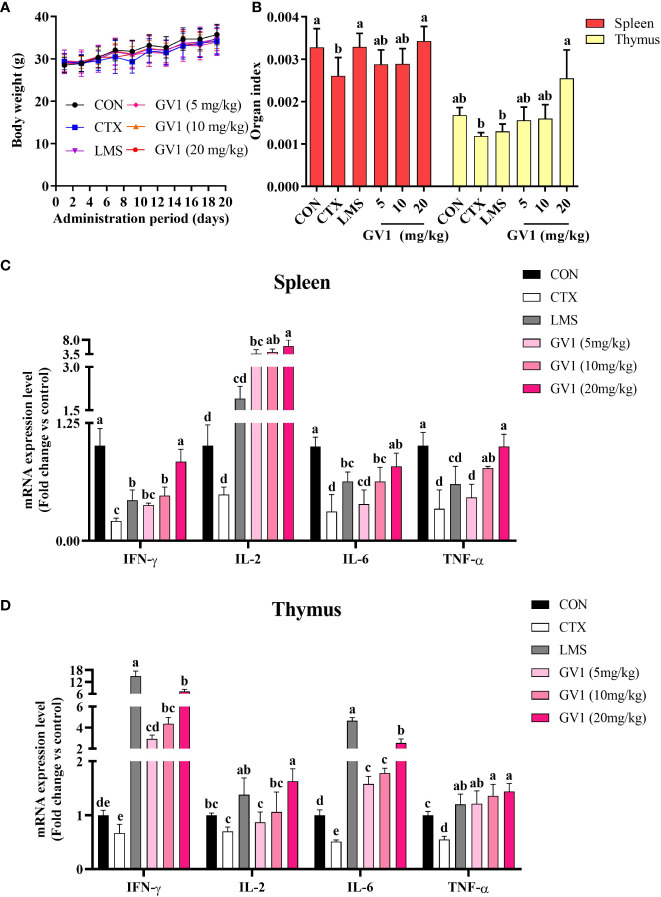
Effects of GV1 on body weight and immune-related organs of CTX-treated immunosuppressed mice. **(A)** Body weight; **(B)** Spleen and thymus indexes; **(C)** Expression of mRNA in spleen; **(D)** Expression of mRNA in thymus. All data are presented as means ± S.D. Adopt the Duncan analysis method. Different letter combinations (a–e) is significant (*p* < 0.05).

*In vitro* experiments indicated that GV1 significantly enhances immunity. Therefore, we investigated the effects of GV1 on CTX-treated immunosuppressed mice. CTX, an alkylating cytotoxic drug, is effective in treating autoimmune disorders and cancer. However, long-term use of high doses of CTX can cause immunosuppression and intestinal problems such as viral infections and microflora disorders (32). Hence, CTX is frequently used to suppress immunity in mouse models. Body weight and immune organ indexes play important roles in the health of mice (33). LMS is a compound that improves immune responses, especially under immunocompromised conditions; hence, it was used as a positive control in this study (3). During the experimental period, body weight slightly increased in all the groups and there was no difference between groups (**Figure 3A**). As shown in **Figure 3B**, the spleen and thymus indexes of the CTX-induced group remarkably decreased compared with those of the control group. GV1 treatment restored the spleen and thymus indexes, and this recovery was greater than that in the LMS-treated group. These data indicate that GV1 could reverse the immune organ atrophy induced by CTX.

Furthermore, we evaluated the effect of GV1 on the expression of mRNA of several immune-associated cytokines such as *IL-6*, *TNF-α*, *IFN-γ*, and *IL-2* in mouse spleen and thymus tissues, respectively. As shown in **Figure 3C**, the cytokine (*TNF-α*, *IL-1β*, *IFN-γ*, and *IL-2*) mRNA levels in the spleen tissue were dramatically suppressed in the CTX group compared with those in the control group. However, the GV1 groups exhibited dose-dependent improvements in cytokine expression, compared with that in the CTX group. Notably, the cytokine mRNA expression in the LMS group was significantly lower than that in the GV1 groups. **Figure 3D** illustrates that CTX remarkably reduced the cytokine (*TNF-α*, *IL-1β*, *IFN-γ*, and *IL-2*) expression levels in thymus tissues compared to those in the control group. In contrast, expression of cytokine mRNA in thymus tissues significantly increased in a dose-dependent manner after oral administration of GV1. These results suggest that GV1 greatly enhanced the expression of mRNA of immune-associated cytokines in spleen and thymus tissues of CTX-treated immunosuppressed mice.

### 3.6 Effect of GV1 on cytokine production and histopathological analysis of spleen in immunosuppressed mice

**Figure 4:**
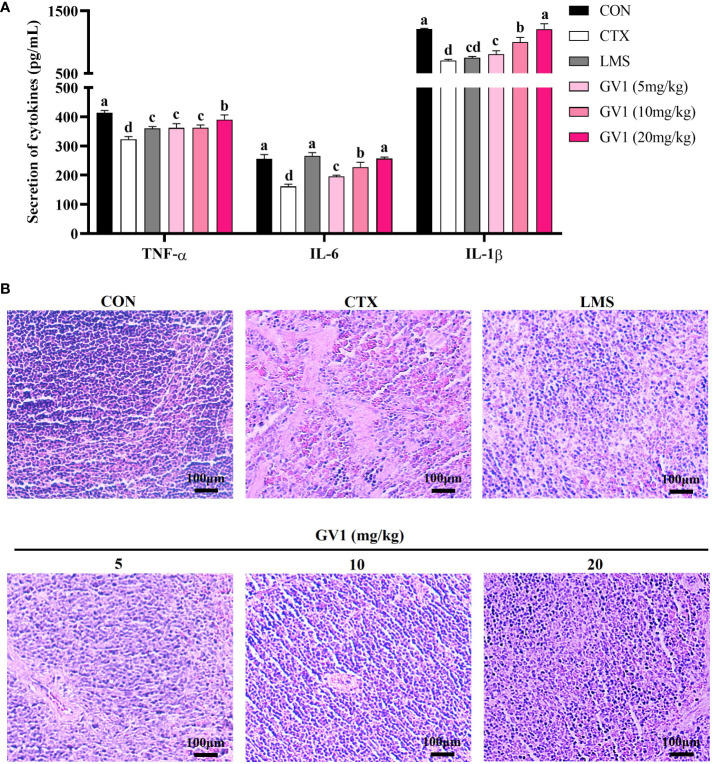
Immunomodulation effects of GV1 on spleen tissue in CTX-treated immunosuppressed mice. **(A)** Secretion of cytokines in spleen; **(B)** Histology of spleen. (100 X, Scale bar = 100μm). All data are presented as means ± S.D. Adopt the Duncan analysis method. Different letter combinations (a–d) is significant (*p* < 0.05).

The spleen is an important immune organ that removes antigens from the blood and initiates innate and adaptive immune responses against pathogens (34). Therefore, we examined the effect of GV1 on cytokine production and conducted a histopathological analysis of the spleen in CTX-treated mice. As shown in **Figure 4A**, CTX treatment suppressed immunocyte action, leading to a decrease in the levels of immune-related cytokines (IL-1β, IL-6, and TNF-α) in spleen tissues of mice. GV1 dramatically increased the secretion of IL-1β, IL-6, and TNF-α in a dose-dependent manner. These findings suggest that GV1 could improve cytokine secretion in the spleen of immunosuppressed mice.

Next, the spleen histology was observed. Compared with those in the control group, the spleen cells in the CTX-treated group were sparse and irregularly arranged (**Figure 4B**). The HE stain histopathological images also showed clear necrotic areas devoid of cell structures and intercellular space dilatation. However, the number of such areas decreased in a dose-dependent manner in the GV1 treatment groups. In particular, at a concentration of 20 mg/kg GV1, the spleen tissues were tightly arranged and dense with clear nuclei as well as less interstitial spaces; thus, their state was better than that in the LMS group. These results demonstrate that GV1 can reverse CTX-induced damage in spleen tissues.

### 3.7 Effect of GV1 on colon histology in immunosuppressed mice

**Figure 5:**
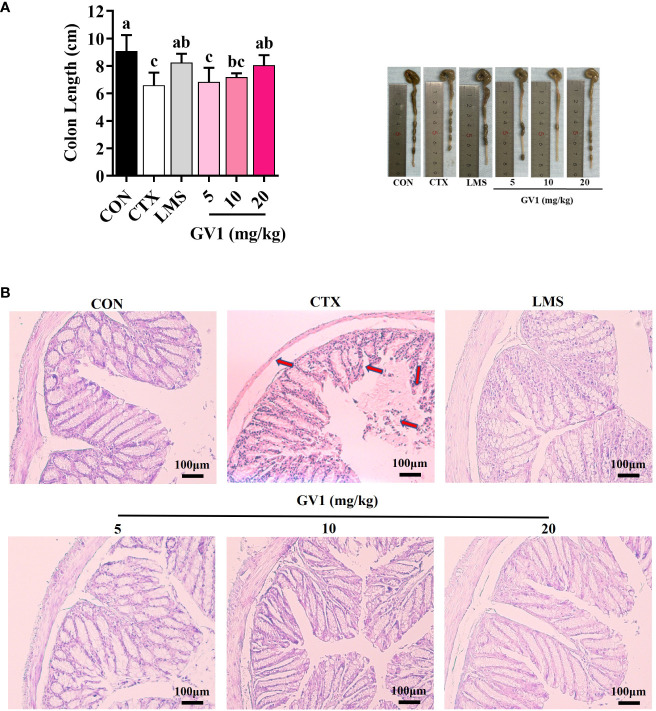
Protective effects of GV1 on colon in CTX-treated immunosuppressed mice. **(A)** Colon length; **(B)** Colon histology. (100 X, Scale bar = 100μm). All data are presented as means ± S.D. Adopt the Duncan analysis method. Different letter combinations (a–c) is significant (*p* < 0.05).

Clinical evidence has shown that CTX treatment can cause colon damage, which hinders gut immunity. Colon length was considerably lower in the CTX group than in the control group (**Figure 5A**); oral administration of GV1 reversed this decrease. At dosages of 10 and 20 mg/kg, GV1 dramatically increased colon length. HE-stained colorectal sections of CTX-treated mice indicated that the thickness of the epithelium had significantly reduced and that inflammatory cells had infiltrated the submucosa and mucosa (**Figure 5B**). In contrast, the GV1 groups exhibited remarkable protection against colonic crypt degradation and tissue inflammation. Interestingly, the colon tissue almost completely recovered upon GV1 treatment at a dosage of 10 mg/kg. Therefore, GV1 restored the length of the colon and reduced colonic damage in a mouse model of CTX-induced immunosuppression.

### 3.8 Effect of GV1 on gut microbiota in immunosuppressed mice

**Figure 6:**
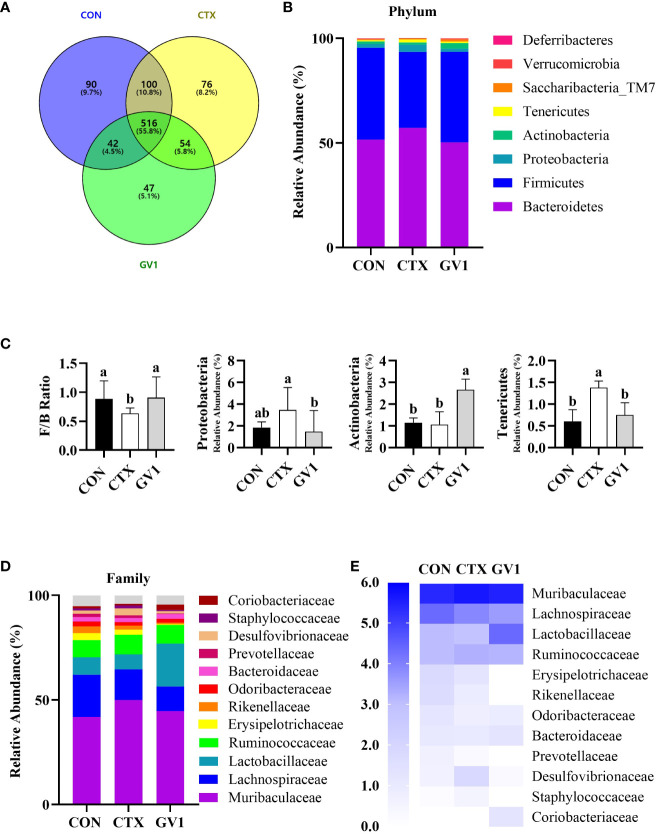
Modulation of gut microbiota composition by GV1 and comparative analysis phylum and family levels in CTX-treated immunosuppressed mice. **(A)** Venn diagram representation of OTUs in CON, CTX, and GV1 groups; **(B)** Relative abundances of gut microbiota in CON, CTX, and GV1 groups at the phylum level, presented as a stacked bar chart. **(C)** *Firmicutes/Bacteroidetes* ratio; Relative abundance of *Proteobacteria*, *Actinobacteria*, and *Tenericutes*; **(D)** Relative abundances of gut microbiota in CON, CTX, and GV1 groups at the family level, presented as a stacked bar chart; **(E)** Heatmap image of family level. All data are presented as means ± S.D. Adopt the Duncan analysis method. Different letter combinations (a, b) is significant (*p* < 0.05).

The modulatory effect of GV1 on gut microbiota was investigated *via* the high-throughput 16S rDNA sequencing of fecal samples. The Venn diagram in **Figure 6A** presents OTUs that were unique and common among three groups; there were a total of 965 OTUs in all the groups. There were 516 shared OTUs (55.8% of the total OTUs) among the groups. The CTX and GV1 groups showed 76 and 47 exclusive OTUs, respectively, whereas the CON group displayed 90 exclusive OTUs. In addition, the diversity and richness analysis, including metrics such as Ace, Chao1, Shannon, and Simpson revealed that GV1 group exhibited lower values in comparison to the CON and CTX groups (**Table 1**).

**Table 1:** α-Diversity metrics of gut microbiota across study groups.

Group	ACE	Chao1	Shannon	Simpson
**CON**	661.7 ± 5.5	652.8 ± 4.86	4.4 ± 0.2	0.032 ± 0.006
**CTX**	650.0 ± 40.2	632.8 ± 38.2	4.3 ± 0.1	0.032 ± 0.004
**GV1**	601.1 ± 36.4	583.3 ± 31.8	4.0 ± 0.3	0.041 ± 0.017

We assessed the relative abundance of species at the phylum and family levels to identify specific taxa related to GV1. The intestines of mice mainly harbor *Firmicutes, Bacteroides, Proteobacteria, Actinobacteria*, and *Tenericutes* (**Figure 6B**). The differences in the relative abundance of these five major phyla were compared. CTX treatment led to a decreased *Firmicutes/Bacteroidetes* ratio and a significant increase in *Proteobacteria* and *Tenericutes* abundance, whereas GV1 treatment increased the abundance of *Actinobacteria*, resulting in a microbiota composition similar to that of the control group (**Figure 6C**). The gut microbiota varied at the family level, with the top 12 relative abundances being exhibited by *Muribaculaceae*, *Lachnospiraceae*, *Lactobacillaceae*, *Ruminococcaceae*, *Erysipelotrichaceae*, *Rikenellaceae*, *Odoribacteraceae*, *Bacteroidaceae*, *Prevotellaceae*, *Desulfovibrionaceae*, *Staphylococcaceae*, and *Coriobacteriaceae* (**Figure 6D**). CTX altered the relative abundance of *Desulfovibrionaceae* and *Staphylococcaceae* compared with that in the control and GV1 groups. GV1 treatment significantly altered the relative abundances of *Lactobacillaceae*, *Bacteroidaceae*, and *Coriobacteriaceae* (**Figure 6E**).

**Figure 7:**
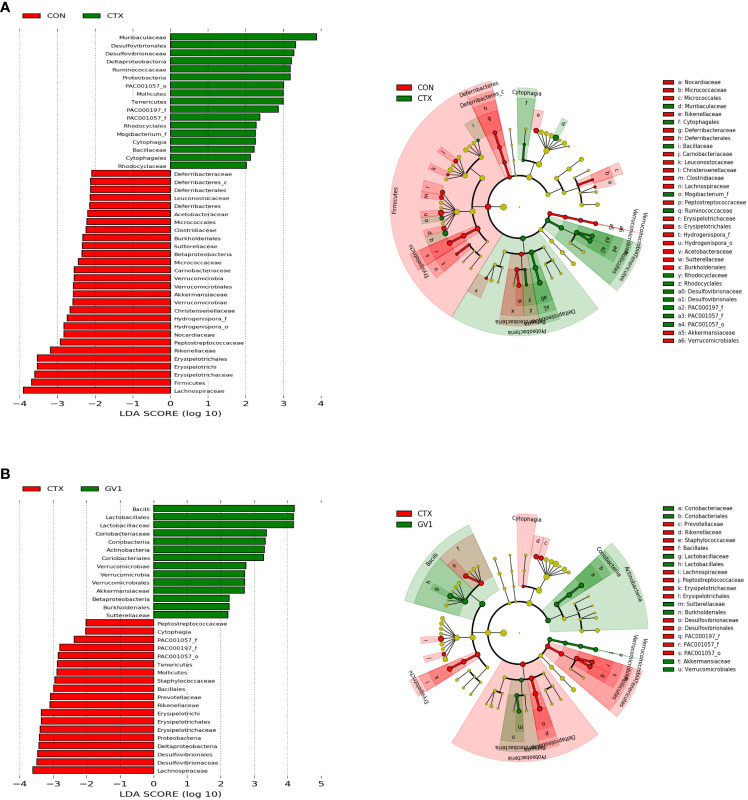
LEfSe analyses of fecal microbes *via* cladogram and histogram based on LDA score. **(A)** CON group *vs*. CTX group; **(B)** CTX group *vs*. GV1 group.

LEfSe was utilized to identify taxa with significant differences in abundance. The linear discriminant analysis (LDA) is a statistical method widely used in multivariate analysis to find the linear combinations of features that best discriminate between different classes. The cladogram visually represents the evolutionary relationships or similarities between different groups, while the histogram provides a graphical representation of the distribution of LDA scores, shedding light on the significance and impact of each component’s abundance in the context of differential effects (35). The LEfSe results of comparing the control and CTX treatment groups (**Figure 7A**) showed that CTX treatment promoted the relative abundance of *Muribaculaceae*, *Proteobacteria*, *Deltaproteobacteria*, *Desulfovibrionales*, *Desulfovibrionaceae*, *Ruminococcaceae*, *Tenericutes*, and *Mollicutes*. In contrast to the CTX treatment (**Figure 7B**), GV1 treatment suppressed the relative abundance of *Lachnospiraceae*, *Proteobacteria*, *Deltaproteobacteria*, *Desulfovibrionales*, *Desulfovibrionaceae*, *Erysipelotrichia*, *Erysipelotrichales, Erysipelotrichaceae*, *Rikenellaceae*, *Prevotellaceae*, *Bacillales*, *Staphylococcaceae*, *Tenericutes*, and *Mollicutes.* Moreover, GV1 treatment promoted the growth of *Bacilli*, *Lactobacillales*, *Lactobacillaceae*, *Actinobacteria*, *Coriobacteriia*, *Coriobacteriales*, *Coriobacteriaceae*, *Verrucomicrobia*, *Verrucomicrobiaceae*, *Verrucomicrobiales*, and *Akkermansiaceae.*

### 3.9 Spearman correlation between gut microbiota and host immune responses

**Figure 8:**
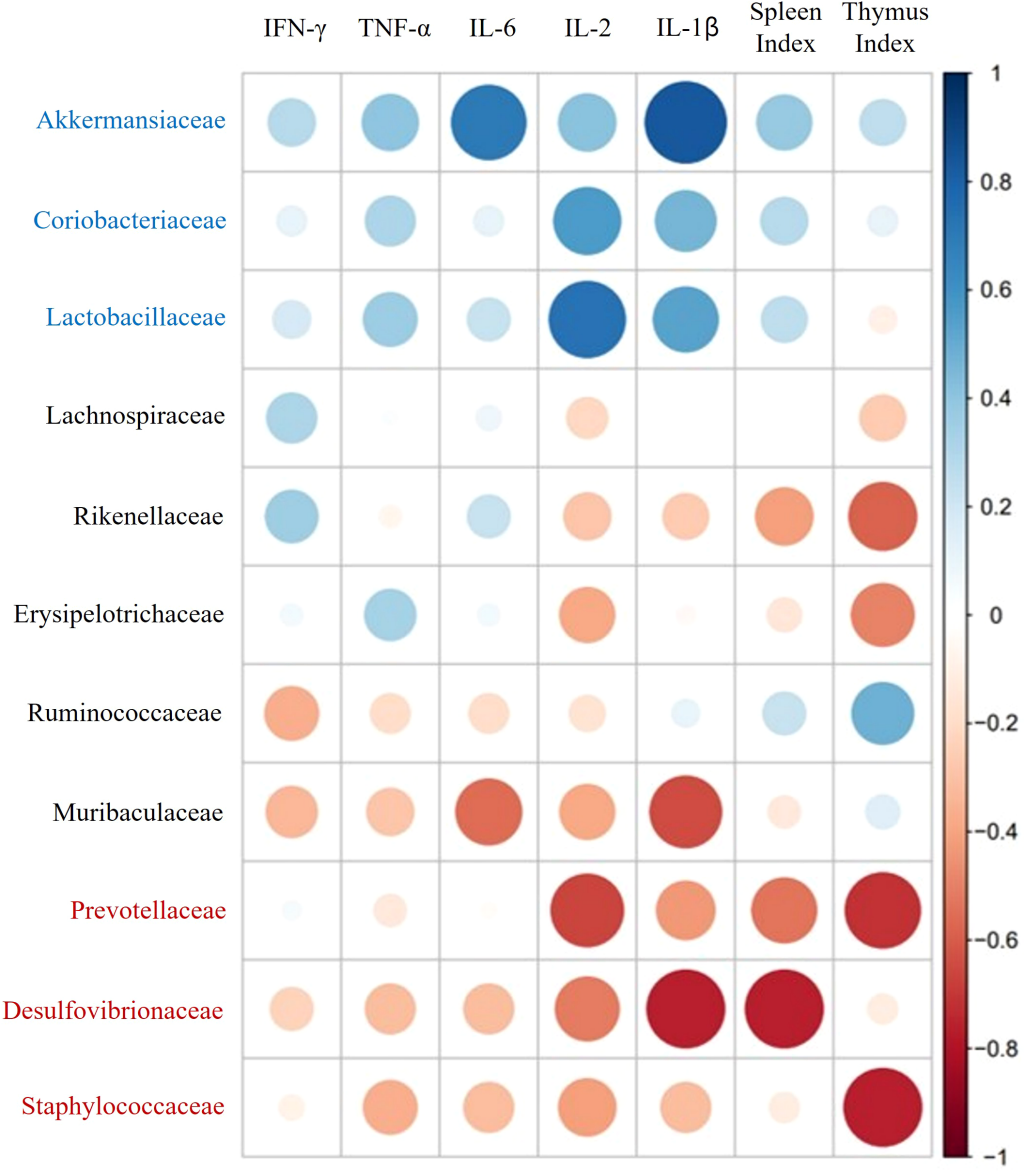
Spearman correlation analysis of immune-related mediators and key microbial communities.

Spearman correlation analysis was used to calculate the correlation coefficient between gut microbial families and immune response mediators. As depicted in **Figure 8**, immune indicators such as immune-related cytokines (IFN-γ, TNF-α, IL-2, IL-6, and IL-1β) and immune organ indexes (spleen and thymus) had highly positive correlations with three types of host relative abundances, namely, those of *Akkermansiaceae*, *Coriobacteriaceae*, and *Lactobacillaceae* in the family level. In contrast, these indicators had strong negative correlations with the relative abundances of *Desulfovibrionaceae*, *Staphylococcaceae*, and *Prevotellaceae.*

## 4 Discussion

Paraprobiotics, also known as inactivated probiotics, have been prepared using several methods, such as heat-killing, sonication, and UV treatment, which not only inactivate microorganisms but also alter their cellular structure (13, 15, 36). The components of probiotic structures have been reported to play an important role in the regulation of immune responses (37). Live cells of *B. velezensis* have been studied extensively for their biological activity; however, the biological effect of inactive *B. velezensis* has not received much attention (38). Therefore, we studied the immunomodulatory effects of heat-killed *B. velezensis* GV1 *in vitro* and *in vivo* to identify the underlying contributing to the beneficial effects of paraprobiotics on immunomodulation.

Macrophages play a crucial role in the immune system by detecting and destroying pathogens. Upon activation, macrophages generate NO, accompanied by the increased expression of *iNOS* gene. NO serves as a signaling molecule that induces interactions among various immune cells to enhance the immune system’s ability to respond to pathogens, inhibiting their replication and thereby, improving overall immune function (17). The increase in NO production and *iNOS* expression in RAW 264.7 macrophages treated with heat-killed GV1 suggests that GV1 enhances immunity (**Figures 2A, B**). Additionally, GV1 dose-dependently increased the expression and secretion of immune-related cytokines such as TNF-α, IL-6, and IL-1β. Macrophages have the ability to release cytokines, which bind to specific receptors on cells and initiate an immune response. The application of heat-treated *Levilactobacillus brevis* KU15159 has demonstrated the stimulation of immune responses which is evidenced in the upregulation of TNF-α, IL-6, IL-1β in RAW 264.7 cells (39). Therefore, we speculated that GV1 may induce immune responses by stimulating the secretion of NO and immune-related cytokines in RAW 264.7 macrophages.

To further characterize the immunity-enhancing effect of GV1, we studied its immunomodulatory activity in the CTX-treated immunosuppressed mouse model. The immune response is suppressed as evidenced by a decrease in the size of the spleen and thymus, along with a reduction in the expression and production of most immune-related cytokines, upon CTX treatment (40). The thymus primarily, oversees the development of T lymphocytes, and the spleen captures pathogens and activates and coordinates the response of immune cells. Therefore, the thymus and spleen play pivotal roles as major lymphoid organs in the immune system, contributing significantly to lymphocyte development and the orchestration of adaptive immune responses (41). As expected, the spleen and thymus indexes significantly decreased upon CTX administration. However, these decreases were reversed upon GV1 administration. Furthermore, GV1 dose-dependently enhanced the expression of *IL-6*, *TNF-α*, *IFN-γ*, and *IL-2* mRNA in the thymus and spleen tissues of immunodeficient mice. Notably, the expression of *IL-2* mRNA greatly increased in spleen tissues in the GV1 groups, compared with that in the LMS group. IL-2 is a multifunctional cytokine that increases NK cell lysis activity, promotes T and B cell proliferation, and activates Treg cells, thus impairing killer cell differentiation (42). These immune-related cells mainly reside in spleen tissues; therefore, the spleen is considered a key immune organ in the body (43). Hence, the protective effects of GV1 on the spleen in CTX-treated immunosuppressed mice were further investigated using ELISA assays and HE staining. Our data show that GV1 remarkably enhances IL- 6, TNF-α, and IL-1β production in the spleen of immunosuppressed mice. Histological results indicate that GV1 can repair CTX-induced spleen tissue damage. Similarly, in our previous study, *Curtobacterium proimmune* K3 lysate significantly stimulated the secretion of immune-related cytokines in the thymus and spleen, and reversed the atrophy of these organs in CTX-treated mice (33). Therefore, we conclude that GV1 can repair immune organ damage and promote the expression of immune-related cytokines to improve the immune system in immunosuppressed mice.

The intestine is a vital organ with key functions in digestion, nutrient absorption, and immunity. The intestinal ecosystem continuously interacts to optimize these functions and maintain the integrity of the gut (44). In conditions of immune suppression and inhibition, the immune system fails to activate adequately, leading to damage in the intestinal tract (45). In this study, we investigated the role of GV1 in improving colon damage caused by immune suppression. Treatment with GV1 demonstrated a protective effect on the colon by increasing its length and alleviating damage. This suggests that GV1 plays a role in ameliorating immune suppression-induced damage, restoring colon function, and enhancing the immune system’s resilience against infections and other immune-related issues. The gut microbial community, especially in relation to intestinal mucosal immunity, plays a crucial role in regulating the host’s innate and adaptive immune systems. Moreover, the efficacy of immunotherapy can vary depending on the composition of the gut microbiota (45). In this study, gut microbiota regulation by GV1 was investigated in CTX-treated mice. The number of OTUs, the diversity and richness index of the GV1 group slightly decreased compared to those of the normal and CTX groups, suggesting that GV1 can modulate the abundance of bacterial species. It is necessary to analyze the dominant gut microbiota at various taxonomic levels (phylum and family) in order to determine the overall differences and similarities between various groups. Our results indicate that *Bacteroidetes* and *Firmicutes* are the most abundant at the phylum level in fecal microbiota, which could be modulated by paraprobiotic GV1. The *Firmicutes/Bacteroidetes* (F/B) ratio is related to the maintenance of homeostasis, and an imbalance in this ratio can cause obesity or inflammatory bowel disease (46). In this study, CTX decreased the relative abundance of *Firmicutes* and increased that of *Bacteroidetes*, causing an imbalance in the F/B ratio. However, this ratio was balanced after oral administration of GV1, similar to the results of a prior study. Notably, GV1 reduced the relative abundance of *Proteobacteria*, which was promoted by CTX treatment. *Proteobacteria* is a major phylum of gram-negative bacteria that includes a large range of pathogenic organisms, including *Helicobacter pylori*, *Escherichia coli*, and *Salmonella* spp (47). Consistent with our results, *Lactobacillus plantarum* BF_15 has been reported to inhibit the growth of *Proteobacteria*, which was promoted by CTX treatment (48). GV1 administration reversed the CTX-induced reduction in gut microbiota diversity and richness possibly by inhibiting pathogenic bacteria (belonging to *Proteobacteria*). At the family level, the relative abundances of *Lactobacillaceae*, *Akkermansiaceae*, and *Coriobacteriaceae* in the GV1 group were higher than those in the CTX group. *Lactobacillaceae* have been reported to have an effect on the immune system and gastrointestinal microbial community of humans (49). *Akkermansiaceae* members exhibit probiotic properties and are inversely related to various diseases, including inflammation, diabetes, obesity, and metabolic disorders (50–53). *Coriobacteriaceae* family members perform important functions within organisms, such as modulating host glucose metabolism in the liver and regulating bile acid and lipid metabolism in the gut (54). In addition to enhancing the relative abundances of beneficial bacterial families, GV1 reduces the abundance of several families such as *Desulfovibrionaceae*, *Erysipelotrichaceae*, *Prevotellaceae*, and *Staphylococcaceae*, compared to that in CTX-treated immunosuppressed mice. Among these families, *Desulfovibrionaceae* are considered as harmful bacteria that can cause mucosal inflammation by inducing toxic hydrogen sulfide to secrete sulfated mucin (55). Kaakoush revealed that *Erysipelotrichaceae* are involved in gastrointestinal inflammatory disorders and that they are particularly found to be abundant in colorectal cancer patients (56). Based on the result (**Figure 8**), Spearman correlation analysis indicate that three types of key microorganisms (*Akkermansiaceae*, *Coriobacteriaceae*, and *Lactobacillaceae*) positively correlate with immune mediators such as immune-related cytokines and immune organ indexes, consistent with previous research (57). These data demonstrate that GV1 can promote immune responses in CTX-treated immunosuppressed mice by modulating gut microbiota dysbiosis.

This study delved into the entire process, from the production of paraprobiotics GV1 to its immunomodulatory effects, along with subsequent gut recovery and microbial community changes (**Supplementary Figure S1**). However, the predominant use of a mouse model as the experimental subject limits direct applicability to humans. Considering the diversity and complexity of the microbial community, additional diverse microbial community analyses and in-depth studies over time are required. Despite these limitations, GV1 demonstrated positive outcomes in enhancing immune function and regulating microbial community imbalance. This suggests promising potential for GV1 in the treatment and prevention of immune-related diseases and disorders. The research provides insights into the immunological and microbiological characteristics of paraprobiotics GV1, indicating its significance as fundamental data for future studies, particularly in the fields of the food industry and health-functional food applications.

## 5 Conclusion

This study focused on the immunomodulatory effects of paraprobiotics, specifically discussing the ability of the heat-treated form of *B. velezensis* GV1 to regulate the microbial community within the human body. GV1 robustly stimulated immune responses by enhancing the expression and secretion of inflammatory cytokines in RAW 264.7 macrophages. Furthermore, GV1 repaired damage to immune organs and increased the expression of immune-related cytokines in immunosuppressed mice induced by CTX. Additionally, GV1 promoted beneficial bacteria and suppressed harmful bacteria, restoring balance to the microbial community in the intestine. These findings indicate, for the first time, that paraprobiotics prepared from *B. velezensis* GV1 can act as a stimulant to enhance immune responses. Such discoveries could form the basis for developing paraprobiotics as functional foods or drugs aimed at improving the immune system. The effects of GV1 should be further investigated through clinical trials, exploring its potential for industrial use as an immunomodulatory agent.

## Data availability statement

The datasets presented in this study can be publicly found in the online repository under the accession BioProject number PRJNA1073872 (http://www.ncbi.nlm.nih.gov/bioproject/1073872).

## Ethics statement

The animal studies were approved by the Animal Care and Use Guidelines of Kyung Hee University (KHGASP-23-046). The studies were conducted in accordance with the local legislation and institutional requirements. Written informed consent was obtained from the owners for the participation of their animals in this study.

## Author contributions

EEJ: Investigation, Writing – review & editing. H-JL: Methodology, Writing – original draft. MTHT: Formal analysis, Writing – review & editing. MHL: Data curation, Writing – review & editing. Y-JK: Supervision, Writing – review & editing.
