# Supplementary material for: Paraprobiotic derived from Bacillus velezensis GV1 improves immune response and gut microbiota composition in cyclophosphamide-treated immunosuppressed mice
Source: Front Immunol. 2024 Feb 22;15:1285063. doi: 10.3389/fimmu.2024.1285063 (PMC10918466; doi:10.3389/fimmu.2024.1285063)
Supplement: Supplementary file 1 [file DataSheet_1.docx]

Supplementary Material

**Paraprobiotic Derived from *Bacillus velezensis* GV1 Improves Immune Response and Gut Microbiota Composition in Cyclophosphamide-Treated Immunosuppressed Mice**

**Hyo-Jun Lee, Thi Hoa My Tran, Ha Minh Le, Elsa Easter Justine, and Yeon-Ju Kim***

*Graduate School of Biotechnology, and College of Life Science, Kyung Hee University, Yongin-si, 17104, Gyeonggi-do, Republic of Korea*

**Hyo-Jun Lee** – Email: gy9707@khu.ac.kr

**Thi Hoa My Tran** – Email**:** hoamytran96@khu.ac.kr

**Ha Minh Le** – Email: lehaminh2311@khu.ac.kr

**Elsa Easter Justine** – Email**:** elsaeaster96@khu.ac.kr

*** Correspondence:**

^*^ Yeon-Ju Kim - *Graduate School of Biotechnology, and College of Life Science, Kyung Hee University, Yongin-si, 17104, Gyeonggi-do, Republic of Korea*.

Email: yeonjukim@khu.ac.kr; Tel: +82-31-201-2645; Fax: +82-31-204-8116.

## Supplementary Figure





**Supplementary Figure S1.** Experimental workflow of paraprobiotics GV1.

## Supplementary Table

| Primer | Sequence (5′-3′) | |
| --- | --- | --- |
| *IL-6* | Forward  Reverse | 5ʹ-GTTCTCTGGGAAATCGTGGA-3ʹ |
|  |  | 5ʹ-TGTACTCCAGGTAGCTATGG-3ʹ |
| *TNF-α* | Forward  Reverse | 5ʹ-AGCCCACGTCGTAGCAAACCAC-3ʹ |
|  |  | 5ʹ-AACACCCATTCCCTTCACAG-3ʹ |
| *IL-1β* | Forward  Reverse | 5ʹ-TGCAGAGTTCCCCAACTGGTAC-3ʹ |
|  |  | 5ʹ-GTGCTGCCTAATGTCCCCTT-3ʹ |
| *IFN-γ* | Forward  Reverse | 5ʹ-TATCTCTTTCTACCTCAGAC-3ʹ |
|  |  | 5ʹ-GCAATCACAGTCTTGGCTAATTAG-3ʹ |
| *IL-2* | Forward  Reverse | 5ʹ-CCTGAGCAGGATGGAGAATTA-3ʹ |
|  |  | 5ʹ-TCCAGAACATGCCGCAGAG-3’ |
| *iNOS* | Forward  Reverse | 5ʹ-AATGGCAACATCAGGTCGGCCA-3ʹ |
|  |  | 5ʹ-GCTGTGTGTCACAGAAGTCT-3ʹ |
| *GAPDH* | Forward  Reverse | 5ʹ-ACCACAGTCCATGCCATCAC-3ʹ |
|  |  | 5ʹ-CCACCACCCTGTTGCTGTAG-3ʹ |

**Supplementary Table S1.** Primer sequences used for qRT-PCR analysis.
